# Unexpected Role of Sterol Synthesis in RNA Stability and Translation in *Leishmania*

**DOI:** 10.3390/biomedicines9060696

**Published:** 2021-06-19

**Authors:** Zemfira N. Karamysheva, Samrat Moitra, Andrea Perez, Sumit Mukherjee, Elena B. Tikhonova, Andrey L. Karamyshev, Kai Zhang

**Affiliations:** 1 Department of Biological Sciences, Texas Tech University, Lubbock, TX 79409, USA; samrat.moitra@ttu.edu (S.M.); andrea.perez@ttu.edu (A.P.); sumit.mukherjee@wustl.edu (S.M.); 2 Honors College, Texas Tech University, Lubbock, TX 79409, USA; 3 Division of Infectious Diseases, Department of Medicine, Washington University School of Medicine, St. Louis, MO 63110, USA; 4 Department of Cell Biology and Biochemistry, Texas Tech University Health Sciences Center, Lubbock, TX 79430, USA; elena.tikhonova@ttuhsc.edu (E.B.T.); andrey.karamyshev@ttuhsc.edu (A.L.K.)

**Keywords:** *Leishmania*, sterol, C-14-demethylase, stress tolerance, RNA degradation, polysome, endoplasmic reticulum, translation, regulation of gene expression

## Abstract

*Leishmania* parasites are trypanosomatid protozoans that cause leishmaniasis affecting millions of people worldwide. Sterols are important components of the plasma and organellar membranes. They also serve as precursors for the synthesis of signaling molecules. Unlike animals, *Leishmania* does not synthesize cholesterol but makes ergostane-based sterols instead. C-14-demethylase is a key enzyme involved in the biosynthesis of sterols and an important drug target. In *Leishmania* parasites, the inactivation of C-14-demethylase leads to multiple defects, including increased plasma membrane fluidity, mitochondrion dysfunction, hypersensitivity to stress and reduced virulence. In this study, we revealed a novel role for sterol synthesis in the maintenance of RNA stability and translation. Sterol alteration in C-14-demethylase knockout mutant leads to increased RNA degradation, reduced translation and impaired heat shock response. Thus, sterol biosynthesis in *Leishmania* plays an unexpected role in global gene regulation.

## 1. Introduction

Protozoan parasites of the genus *Leishmania* cause leishmaniasis infecting 10–12 million people worldwide [1]. There are three major forms of leishmaniasis. Visceral leishmaniasis is the most severe form, with a mortality rate of almost 100% if left untreated [2,3]. Mucocutaneous leishmaniasis can produce disfiguring lesions of the nose, mouth and throat cavities. The cutaneous form of leishmaniasis is the most common type representing 50–75% of all new cases [4].

All three forms of leishmaniasis are transmitted through the bite of sand fly vectors (*Phlebotomus* spp. and *Lutzomyia* spp.). During their life cycle, these dixenic protozoans alternate between flagellated, extracellular promastigotes, which live in the midgut of sand flies, and non-flagellated amastigotes residing in the phagolysosomal compartment of mammalian macrophages [5]. Promastigotes are transmitted with sand fly saliva into the mammalian host during blood feeding, where they are rapidly engulfed by phagocytic cells and differentiate into amastigotes. The changes in temperature, pH and nutrients that *Leishmania* parasites encounter in the mammalian host appear to be essential for the promastigote to amastigote differentiation [6]. Options for leishmaniasis control are very limited due to the lack of a vaccine, toxic side effects of drugs and rapid emergence of drug-resistant strains [7,8]. Therefore, there is an urgent need to understand the molecular mechanisms utilized by *Leishmania* parasites to survive in different hosts, as discoveries in basic biology can lead to new medicine.

Lipid metabolism is a very important yet understudied area in protozoan parasites. Besides serving as an energy source and building blocks of the membrane, lipids are implicated in parasite-host interaction and pathogenesis [9,10,11]. Drugs such as miltefosine and antimony induce lipid perturbations which contribute to the development of drug resistance in *Leishmania* parasites [12,13]. Notably, *Leishmania* parasites produce different types of sterols from humans, making the sterol synthesis pathway an attractive pharmacological target [14,15]. Specifically, *Leishmania* produces ergostane-based sterols such as ergosterol and 5-dehydroepisterol while human cells synthesize cholesterol [16]. Sterols are important constituents of the plasma membrane (PM), endoplasmic reticulum (ER) and organellar membranes. Because of their rigid and hydrophobic structure, sterols reduce the flexibility of acyl chains of neighboring phospholipids and increase membrane rigidity and tightness [17]. They are also involved in vesicular transport [17]. Sterols defects are known to affect not only membrane permeability and fluidity but also the localization of membrane-bound proteins and transport of proteins [18]. In mammalian cells, the distribution of sterols in specific organellar membranes is strictly regulated, and its impairment leads to many diseases [19]. In yeasts, defects in sterol synthesis lead to disruption in ER organization and changes in lipid organization of the PM [20].

C-14-demethylase (C14DM) catalyzes the removal of a methyl group from the carbon-14 position of lanosterol, a key step in the synthesis of ergostane-based sterols [21]. The C14DM-null mutant (*c14dm^−^*) has been characterized in *Leishmania major* LV39 strain [22]. This mutant cannot remove the C-14-methyl group from lanosterol or other sterol intermediates. The defect leads to increased membrane fluidity, mitochondrion dysfunction, superoxide accumulation, hypersensitivity to heat and severely reduced virulence in mice [22,23].

In this study, we revealed an unexpected role of C14DM in the regulation of RNA levels in *Leishmania* parasites. We uncovered that defects in sterol synthesis lead to reduced RNA stability and protein synthesis, which likely contribute to their impaired stress response. Our study is the first of its kind that links sterol synthesis to the global regulation of gene expression at the level of RNA stability.

## 2. Materials and Methods

### 2.1. Reagents

Actinomycin D and MitoSox Red were purchased from Sigma (St. Louis, MO, USA) and Thermo Fisher Scientific (Waltham, MA, USA), respectively. Trizol and Trizol LS were purchased from Life Technologies (Carlsbad, CA, USA). L-Glutathione (reduced form) and antimycin A were purchased from ENZO Life Sciences (Farmingdale, NY, USA). All other chemicals were purchased from VWR International (Radnor, PA, USA) unless otherwise specified.

### 2.2. Leishmania Culturing and Treatments

*Leishmania major* LV39 (Rho/SU/59/P), *c14dm^−^*(*C14DM*-null mutant) and *c14dm^−^/* + C14DM (episomal add-back) promastigotes were cultivated at 27 °C in complete M199 media containing 10% fetal bovine serum and additional supplements as previously described [24]. Culture densities over time were determined by direct cell counting using a hemacytometer. The BCA protein assay kit (Thermo Fisher Scientific) was used to determine protein concentration in cell lysates according to the manufacturer’s recommendation. In order to block transcription and monitor RNA degradation, actinomycin D was added at 10 μg/mL to mid-log phase cultures (3–6 × 10^6^ cells/mL). Equal aliquots of cultures were taken at the indicated times for further analysis.

Some experiments required antioxidant treatment of cells. Briefly, *Leishmania* promastigotes were seeded at 4 × 10^5^ cells/mL and treated with different concentrations of L-glutathione (1, 2 or 4 mM) for 48 h prior to cell collection for further analysis. In order to induce mitochondrial oxidative stress, LV39 wild-type (WT) cells prepared at a concentration of 1.0 × 10^7^ cells/mL were treated with 5 µM of antimycin A for 3 h at 27 °C. Then parasites were stained with 10 μM of MitoSox Red. Mean fluorescence intensities (MFI) were determined by flow cytometry using an Attune NxT Acoustic Flow Cytometer (Thermo Fisher Scientific) to confirm the induction of mitochondrial stress prior to cell collection for downstream analysis. Cell viability was determined by measuring the incorporation of propidium iodide (PI, 5.5 µg/mL) via flow cytometry as described [23]. Neither antimycin A nor L-glutathione treatments significantly affected cell viability based on PI staining (<5% PI positive).

### 2.3. Polysome Profiling

Polysome profiling experiments were performed as published earlier [25]. Briefly, *Leishmania* promastigotes of WT, *c14dm^−^* and *c14dm^−^/* + C14DM were grown in flasks until cell density reached 5 × 10^6^ cells/mL. An equal number of cells (1.5 × 10^8^ promastigotes) were used for polysome profiling for each experimental condition. Before lysis, cells were treated with 100 µg/mL of cycloheximide for 15 min at 27 °C to stabilize ribosomes on mRNAs. Cells were lysed on ice in a buffer containing 20 mM Hepes–KOH (pH 7.5), 10 mM MgCl_2_, 100 mM KCl, 2 mM DTT, 1% NP-40, 1 × protease inhibitor cocktail (EDTA-free) from Sigma, 200 units/mL RNasin (Thermo Fisher Scientific) and 100 µg/mL of cycloheximide. Details of cell lysate preparation were described previously [25]. The lysate was clarified by centrifugation at 11,200 g for 10 min at 4 °C. In order to separate polysomes, 500 µL of the clarified lysate was loaded on top of a 10%–50% sucrose gradient containing 20 mM Hepes–KOH (pH 7.5), 100 mM KCl, 10 mM MgCl_2_, 1 mM DTT and 200 units/mL RNasin, and subjected to ultracentrifugation using an SW41 rotor for 2 h at 260,000× *g* and 4 °C. After centrifugation, 500 µL of fractions were collected using a Piston Gradient Fractionator from BioComp Instruments (Fredericton, NB, Canada). Trizol LS was added to fractions immediately, and samples were stored at −80 °C until RNA extraction.

### 2.4. RNA Extraction, cDNA Preparation and Real-Time Reverse-Transcription Quantitative PCR (RT-qPCR)

Total RNA was isolated using Trizol or Trizol LS (for polysomal fractions) and quantified spectrophotometrically using a NanoDrop device (Thermo Fisher Scientific) as described [25]. cDNA samples were prepared using a High-Capacity cDNA Reverse Transcription Kit from Applied Biosystems (Waltham, MA, USA) according to the manufacturer’s recommendation. Real-time quantitative polymerase chain reactions (RT-qPCR) were performed on a Quant Studio 12 K Flex Real-Time PCR System using Power SYBR Green PCR Master Mix (Applied Biosystems), according to the manufacturer’s protocol. The comparative ΔΔCT method was used to quantify the qPCR results [26]. For the analysis of gene expression, synthetic outer membrane protein A (OmpA) mRNA was added to all samples prior to RNA extraction and used for normalization as described [25]. The RNA levels were analyzed by RT-qPCR for the following genes: tubulin, heat shock protein 70 (HSP70), heat shock protein 83 (HSP83), sterol-24-C- methyltransferase (SMT), 18S ribosomal RNA (18S rRNA) and 28S ribosomal RNA (28S rRNA). The primers used in RT-qPCR reactions and corresponding gene identification numbers are presented in Supplementary File S1.

### 2.5. ER Labelling and Confocal Microscopy

ER staining was performed using an anti-*Trypanosoma brucei* BiP antibody (kind gift from Dr. Jay Bangs, University at Buffalo, SUNY). Log phase WT, *c14dm^−^* and *c14dm^−^/* + C14DM promastigotes were incubated at 27 °C (control) or 37 °C (experimental) for 2 h. Afterwards, parasites were washed in phosphate-buffered saline (PBS), attached to poly-L-lysine coated coverslips, fixed with 3.7% formaldehyde, and then permeabilized on ice with ethanol. Incubation with the rabbit anti-*T*. *brucei* BiP antiserum (1:1000) was performed at room temperature for 40 minutes. After washing with PBS, coverslips were incubated with a goat anti-rabbit-Alexa Fluor 488 (1:2000) antiserum for 40 min. An Olympus (Center Valley, PA, USA) Fluoview FV3000 Laser Scanning Confocal Microscope was used to visualize the intensity and localization of BiP from randomly selected cells using the cellSens Imaging Software (Olympus). The number of cells analyzed at 27 °C was 63 (WT), 131 (*c14dm^−^*) and 95 (*c14dm^−^/* + C14DM). The number of cells analyzed at 37 °C was 152 (WT), 106 (*c14dm^−^*) and 65 (*c14dm^−^/* + C14DM).

### 2.6. Statistical Analysis

Unless specified otherwise, assay values in all figures are averaged from three independent biological repeats, and error bars represent standard deviations. The Student’s *t*-test was used in pairwise comparisons. Differences among multiple groups were assessed by One-way Anova followed by Tukey’s or Dunnett’s test. *p*-values indicating statistical significance were grouped in figures as ns: not significant; *: *p* < 0.05; **: *p* < 0.01; and ***: *p* < 0.001.

## 3. Results

### 3.1. Defects in Sterol Synthesis Lead to Global Reduction in RNA and Protein Levels in c14dm^−^

C14DM is an important enzyme in ergosterol biosynthesis and the target of azole drugs. In *L. major*, this enzyme is mostly localized at the ER, and only a minor amount is found in the mitochondrion [22]. Genetic or chemical inactivation of C14DM led to a complete loss of ergostane-based sterols and accumulation of C-14-methylated sterols [22]. *c14dm^−^* mutants also displayed increased membrane fluidity, reduced virulence and extreme sensitivity to stress.

**Figure 1:**
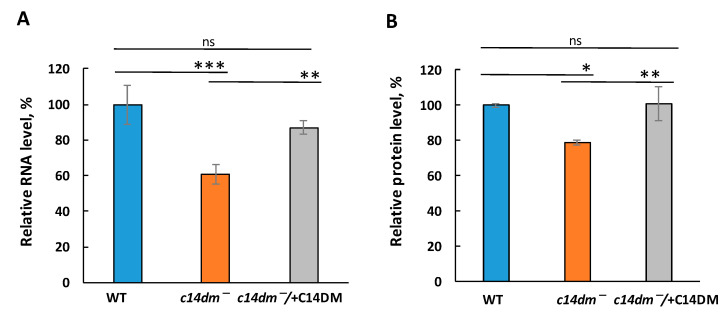
The total RNA and protein levels are reduced in *c14dm*^−^ mutant. (**A**) The total RNA was extracted from 1 × 10^7^ cells of WT, *c14dm^−^* and *c14dm^−^/* + C14DM cells and quantified using NanoDrop in relation to WT. (**B**) The total protein levels from 1 × 10^7^ cells of WT, *c14dm^−^* and *c14dm^−^/* + C14DM were measured using BCA assay, and their relative levels were calculated in relation to WT. ns: not significant; *: *p* < 0.05; **: *p* < 0.01; and ***: *p* < 0.001.

In order to fully understand the consequences caused by defective sterol synthesis, we examined the total RNA and protein levels in *c14dm^−^.* Remarkably, we found an approximately 40% reduction in total RNA levels in *c14dm^−^* in comparison to WT and add-back parasites (Figure 1A). The mutant also had ~20% less total protein (Figure 1B). These results suggest a global dysregulation of gene expression in the cells defective in sterol production. Adding *C14DM* back completely rescues the defects at both RNA and protein levels.

**Figure 2:**
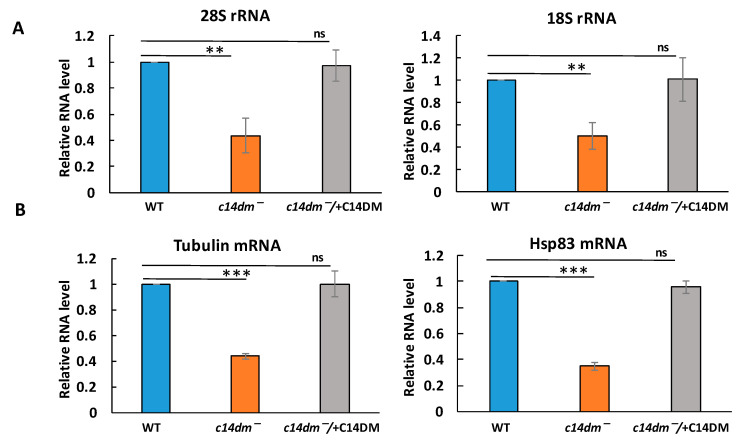
Levels of ribosomal RNA and individual mRNAs are reduced in *c14dm^−^*. The total RNA was extracted from 1 × 10^7^ cells of WT, *c14dm^−^* and *c14dm^−^/* + C14DM cells. The artificial outer membrane protein A (OmpA) mRNA was added prior to RNA extraction for further normalization. RNA levels were measured by RT-qPCR. (**A**) 28S (left panel) and 18S (right panel) rRNA levels. (**B**) Tubulin (left) and HSP83 (right) mRNA levels. ns: not significant; **: *p* < 0.01; and ***: *p* < 0.001.

In eukaryotic cells, most RNA is rRNA, while the protein-encoding mRNA constitutes about 5% of total RNA [27,28]. Global reduction in RNA level suggests that rRNA is likely reduced. In the next experiment, we examined what type of RNA is affected by defects in sterol synthesis. Total RNA was extracted, and RT-qPCR was performed to measure levels of selected RNAs as described [25]. We found that both 28S and 18S rRNA, as well as individual mRNAs for tubulin and HSP83, displayed substantially lower levels in *c14dm^−^* and *c14dm^−^/* + C14DM rescued the defects (Figure 2).

These results suggest that impairment in sterol biosynthesis causes global downregulation of rRNA and mRNAs in *Leishmania major* parasites.

### 3.2. RNA Stability Is Compromised in c14dm^−^

**Figure 3:**
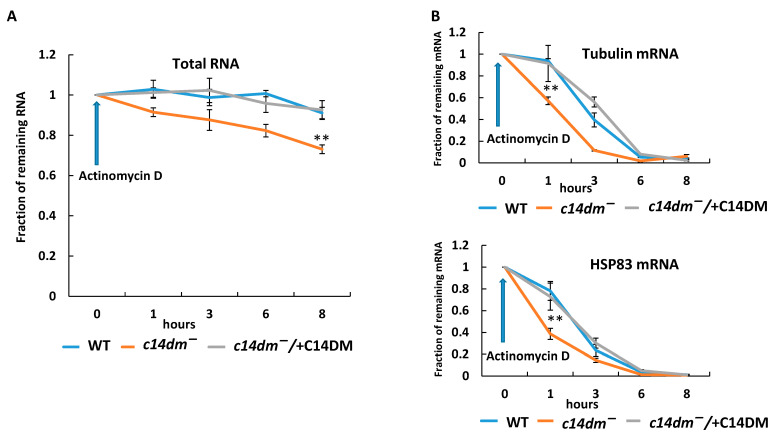
RNA in *c14dm^−^* degrades faster in comparison to WT and add-back parasites. Actinomycin D was added to mid-log cultures, and equal aliquots of cultures were taken at the indicated times. RNA was extracted and analyzed by RT-qPCR relative to the values at the starting point. (**A**) Total RNA was quantified using NanoDrop over time. (**B**) The relative abundance of tubulin and HSP83 mRNA was quantified by RT-qPCR over time. **: *p* < 0.01.

The reduction in RNA levels can be caused either by increased RNA degradation or defects in transcription. In order to examine whether defects in sterol synthesis affect RNA stability, we treated WT, *c14dm^−^* and *c14dm^−^/* + C14DM cells with actinomycin D to block transcription and monitored the rates of RNA degradation. Actinomycin D inhibits all transcription independent of the types of RNA polymerases [29]. Actinomycin D was added to mid-log phase cells, and a time-course experiment was performed as described in Figure 3. The RNA levels were quantified relative to the starting point of actinomycin D treatment for each strain. The absolute amount of total RNA was lower in *c14dm^−^* at the starting point (Figure 1B). Our results demonstrate that total RNA decays faster in *c14dm^−^* after blocking transcription with actinomycin D, providing strong support to the idea that RNA stability is substantially compromised in the mutant (Figure 3A). We checked the rate of degradation of individual mRNAs as well (Figure 3B). Tubulin mRNA decays much faster in the mutant; after one hour of treatment, only about 57% of RNA remains in *c14dm^−^* versus 94% in WT parasites. HSP83 mRNA exhibits even faster degradation. The half-life of HSP83 mRNA is 48.9 min in *c14dm^−^*, 137.7 min in WT and 110.5 min in *c14dm^−^/* + C14DM parasites, respectively (Figure 3B).

We also examined the degradation of 18S and 28S rRNA. While rRNA is considered to be more stable, it is also subject to quality control in eukaryotes [30]. It also degrades under conditions of stress or starvation [31]. We have found that 18S and 28S rRNA degrades significantly faster in the mutant, although they are more stable than tubulin and HSP83 mRNAs (Figure S1).

### 3.3. RNA Degrades Faster during Heat Shock and the Induction of Heat Shock Response Is Compromised in c14dm^−^

**Figure 4:**
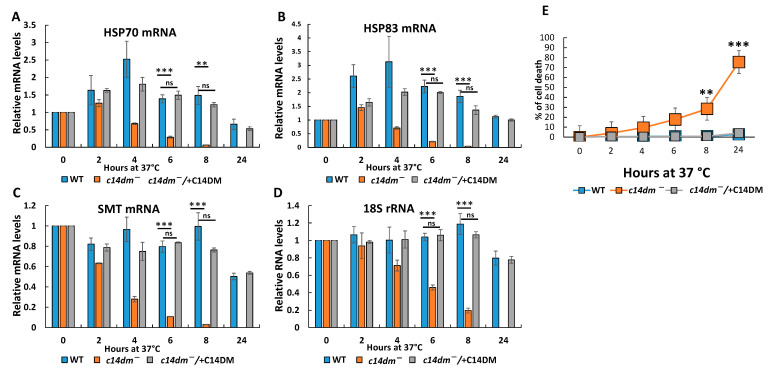
*C14dm^−^* mutants show a dramatic decrease in RNA levels during heat shock. Promastigotes were incubated at 37 °C for 0–24 h, and cells were collected and analyzed at the indicated times. Artificial OmpA mRNA was added prior to RNA extraction for normalization. HSP70 (**A**), HSP83 (**B**), SMT (**C**) mRNA and 18S rRNA (**D**) levels were measured by RT-qPCR. (**E**) Cell viability was monitored by PI staining during heat shock. ns: not significant; **: *p* < 0.01; and ***: *p* < 0.001.

When cells face stress, such as heat shock, a global downregulation of gene expression occurs, but genes involved in stress response to increase survival are selectively upregulated [32]. HSP83 is known to be upregulated in response to heat shock [32]. The *c14dm^−^* mutant displays extreme sensitivity to stress conditions such as heat shock and starvation [22,23]. The ability to cope with stress is essential for *Leishmania* survival in mammals where they encounter dramatic changes in temperature, nutrient availability and pH [33]. To examine if the heat shock response is altered in *c14dm^−^*, we subjected promastigotes to 37 °C treatment and measured HSP70 and HSP83 mRNA levels by RT-qPCR (Figure 4A,B). Sterol-24-C-methyltransferase (SMT) mRNA and 18S rRNA were included as negative controls, which should not be induced under heat shock (Figure 4C,D). As expected, HSP70 and HSP83 mRNAs showed a 2–4-fold increase in WT parasites after four hours at 37 °C. In contrast, *c14dm^−^* mutant had a substantially diminished response (1.2–1.5-fold increase after two hours) to the heat shock. This initial increase was fast and significantly dropped down with longer incubation. The defects were completely reversed in *c14dm^−^/* + C14DM cells (Figure 4A,B). We also observed accelerated degradation of SMT mRNA and 18S rRNA in *c14dm^−^* (Figure 4C,D). After 8 h of incubation at 37 °C, SMT mRNA level was dramatically diminished to ~5% in the mutant, whereas only slight changes were detected in WT and *c14dm^−^/* + C14DM parasites. While 18S rRNA remained stable in WT and add-back parasites even after 8 h of heat shock, its level was strikingly reduced to 18% in sterol defective mutant. As previously described [22], *c14dm^−^* parasites were mostly dead after 24 h of heat shock, while both WT and *c14dm^−^/* + C14DM parasites were mostly alive by that time and maintained 18S rRNA level at around 80% (Figure 4D,E).

These results suggest that in addition to increased membrane fluidity and accumulation of superoxide, a defective heat shock response (failure to properly induce HSP gene expression and accelerated degradation of other RNAs) contributes to the extreme heat sensitivity displayed by *c14dm^−^* (Figure 4E) [22,23].

### 3.4. Reduced RNA Levels in c14dm^−^ Is Not Caused by the Accumulation of Oxidants

**Figure 5:**
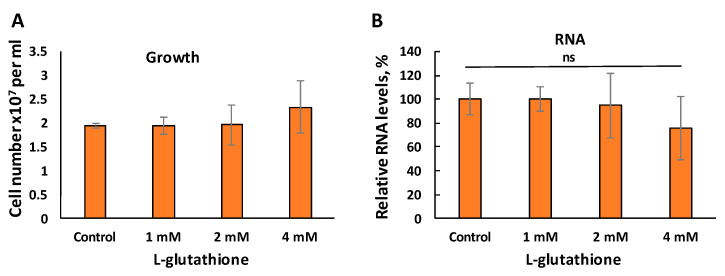
Antioxidant treatment of *c14dm^−^* mutant does not restore RNA levels. (**A**) *C14dm^−^* cells were inoculated at 4 × 10^5^ cells/mL and treated with 1–4 mM of L-glutathione, and culture densities were determined after 48 h. (**B**) Total RNA was extracted from 1 × 10^7^ *c14dm^−^* cells after L-glutathione treatment and quantified by NanoDrop. Percentages represent RNA levels relative to the untreated control. ns: not significant.

*C14dm^−^* cells have elevated levels of reactive oxygen species (ROS), mostly in the form of mitochondrial superoxide [23]. In order to examine if oxidative stress contributes to the RNA reduction, we cultivated *c14dm^−^* cells in the presence of L-glutathione and performed total RNA extractions. Cell growth rates and survival (by PI staining) were similar between control and L-glutathione-treated cells (Figure 5A). Importantly, L-glutathione treatment did not restore the RNA levels in *c14dm^−^* (Figure 5B), although it could partially alleviate the ROS accumulation [23].

**Figure 6:**
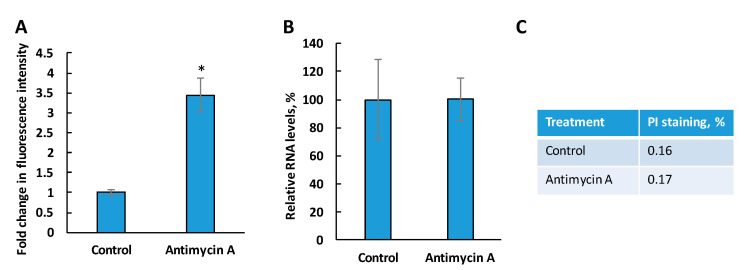
Mitochondrial oxidative stress induced by antimycin A does not reduce RNA levels in WT parasites. (**A**) WT parasites were treated with antimycin A for 3 h at 27 °C and stained with 10 μM of MitoSox Red. Mean fluorescence intensities were determined by flow cytometry. Relative mitochondrial ROS levels were plotted in comparison to untreated WT control cells. (**B**) Total RNA was extracted from 1 × 10^7^ WT cells after antimycin A treatment and quantified. Percentages represent RNA levels relative to the untreated control. (**C**) Cell death was measured by PI staining. *: *p* < 0.05.

In addition, *L. major* WT cells were treated with antimycin A to induce oxidative stress in the mitochondria, and total RNA was extracted from an equal number of cells. While mitochondrial superoxide was induced as expected based on MitoSox staining (Figure 6A) [23], it did not affect the RNA levels (Figure 6B). Cell death was negligible in control and antimycin-treated cells (Figure 6C).

Thus, our data demonstrate that mitochondrial ROS stress is not responsible for the reduction of RNA levels in *c14dm^−^* mutant.

### 3.5. Polysome Profiling Reveals Defects in Translation in c14dm^−^

**Figure 7:**
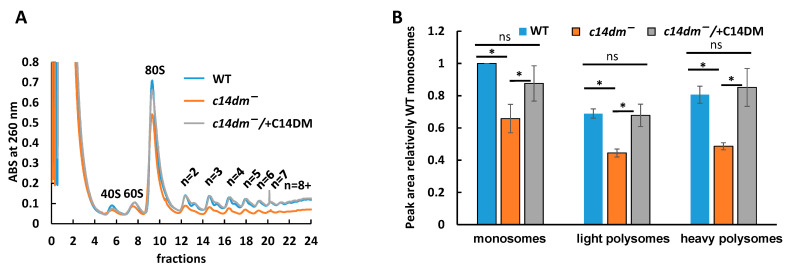
*C14dm^−^* mutant displays reduced monosomal and polysomal peaks. (**A**) Polysomal profile of WT (blue), *c14dm^−^* (orange) and *c14dm^−^/* + C14DM (grey). Ribosomal subunits and polysomes are indicated based on absorbance at 260 nm. The experiment was conducted in four biological repeats, and one representative profile is shown here. (**B**) Quantification of the results shown in (**A**). Areas for peaks of monosomal fractions (80S), the light polysomes (*n* = 2–4) and heavy polysomes (*n* = 5–8+) were calculated using trapezoidal sum (https://bit.ly/36YJVo1 (accessed on 12 July 2020)). Peak areas were then normalized to the WT monosomes. ns: not significant; *: *p* < 0.05.

Reduced mRNA and rRNA levels support the hypothesis that mRNA translation could be compromised in the *c14dm^−^* mutant. To test this notion, a polysome profiling experiment was performed, which is based on monosome and polysome separation in a sucrose gradient. mRNA can be translated by more than one ribosome leading to the formation of light and heavy polysomes. A higher association of mRNAs with light and heavy polysomes supports more efficient translation, while a higher association with monosomes indicates poor translation or translational arrest, as we demonstrated earlier [25]. An equal number of cells from WT, *c14dm^−^* and *c14dm^−^*/ + C14DM were used for polysome profiling. Transcripts were stalled on ribosomes with cycloheximide [34]. Then mRNAs containing a different number of ribosomes were separated by sucrose gradient [35]. Although an equal number of cells were used in each polysome profiling, *c14dm^−^* mutant displays substantially reduced ribosomal peaks (Figure 7A). The heights of both monosome and polysome peaks were 30%–40% lower than WT and *c14dm^−^/* + C14DM parasites, indicating overall reduced engagement of ribosomes in translation (Figure 7B).

**Figure 8:**
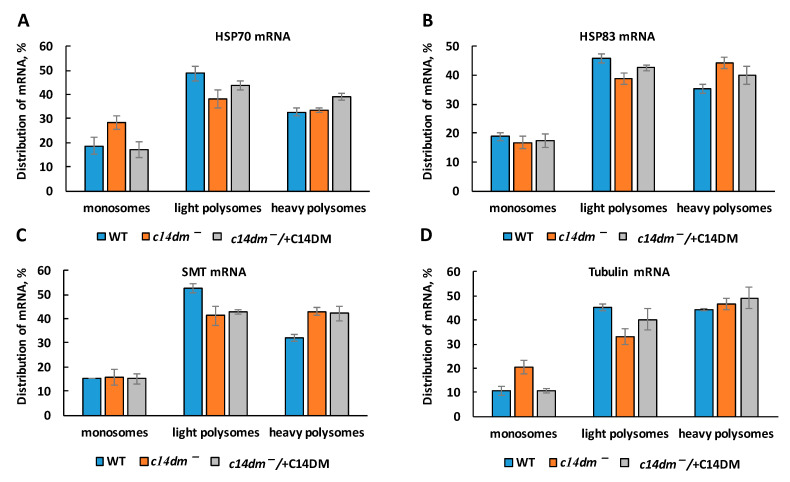
The association of individual mRNAs with monosomes and light and heavy polysomes is largely unaffected in *c14dm^−^* mutant. Distribution of HSP70 (**A**), HSP83 (**B**), SMT (**C**) and tubulin (**D**) mRNAs among monosomes, light polysomes and heavy polysomes was determined by RT-qPCR after RNA extraction from polysomal fractions.

Next, we examined if the association of individual mRNAs with monosomes and light and heavy polysomes were affected in the *c14dm^−^* mutant. We expected two scenarios here: first, the association of all mRNAs with ribosomes was consistently reduced similar to the reduction in steady-state mRNA levels; alternatively, the association with ribosomes for some mRNAs could be affected more than others, leading to a global gene dysregulation instead of proportional reduction in protein production. Lower association of mRNAs with light and heavy polysomes is indicative of less efficient translation, and reductions in polysome peak heights suggested that individual RNAs could be translated less efficiently as well. Our RT-qPCR results, based on four mRNAs, demonstrate the degree of individual mRNA’ association with monosomes, and light and heavy polysomes were largely unaffected by *C14DM*-deletion, supporting a mostly proportional reduction in protein production (Figure 8). We observed a minor increase in association with monosomes for HSP70 mRNA in *c14dm^−^*, but the difference was not statistically significant.

### 3.6. Intensity of ER Staining Is Compromised in c14dm^−^ under Heat Shock

**Figure 9:**
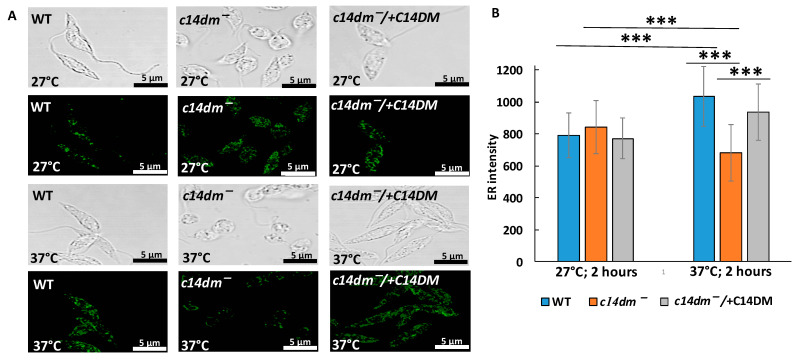
Heat treatment leads to a significant reduction in ER staining in *c14dm^−^*. (**A**) Representative images of WT, *c14dm^−^* and *c14dm^−^/* + C14DM cells are shown. BiP staining was performed after 2 h of incubation at 27 or 37°C. (**B**) Quantitative analysis of BiP-staining intensity. ***: *p* < 0.001.

Sterol is present in both PM and organellar membranes [36]. Rough ER carries ribosomes and is where the majority of translation takes place [37]. Perturbations in sterol synthesis may alter the structure of ER and affect the recruitment of ribosomes for translation. To examine the ER in promastigotes, WT, *c14dm^−^* and *c14dm^−^*/ + C14DM parasites were grown at 27 °C (insect vector temperature) and exposed to 37 °C for two hours (mammalian temperature) followed by staining with the antibody against BiP, an ER protein (Figure 9). Our results indicate that BiP staining at 27 °C was comparable between WT and *c14dm^−^*. However, the 2-h treatment at 37 °C led to a dramatic reduction (~30%) in *c14dm^−^,* suggesting an altered ER at higher temperatures.

## 4. Discussion

Lipid homeostasis is critical for the proliferation and pathogenesis of *Leishmania* parasites, and changes in lipid composition can lead to multiple defects [22,23,38,39,40,41,42,43]. Despite the progress in understanding many lipid pathways, the role of sterol synthesis is not fully defined in protozoan parasites. In contrast to mammalian cells that synthesize cholesterol as the main sterol, *Leishmania* parasites primarily make ergosterol and 5-dehydroepisterol [44,45,46]. C14DM is responsible for the removal of a C-14-methyl group from sterol intermediates. Genetic deletion of this key enzyme leads to the loss of ergostane-based sterols and the accumulation of C-14-methylated sterol intermediates [22]. These defects cause increased PM fluidity and permeability. The presence of sterols is required not only in the PM but also in the membranes of intracellular organelles and lipid droplets [47]. The lack of proper sterols in *c14dm^−^* leads to altered mitochondrial morphology, accumulation of ROS and impairment in respiration [23].

In this study, we revealed an unexpected role of sterol synthesis in RNA stability and translation. First, we found that both total RNA and protein levels are substantially reduced in *c14dm^−^* mutants (Figure 1). Then, we demonstrated that all tested RNAs, including 18S rRNA, 28S rRNA, tubulin and HSP83 mRNAs, display 50%–60% reduction in steady-state RNA levels in comparison to WT and add-back parasites when extracted from an equal number of cells (Figure 2). Next, actinomycin D treatment experiments demonstrated that both mRNA and rRNA are much less stable in the mutant and adding the *C14DM* gene back rescues this defect (Figure 3). In *c14dm^−^*, RNA instability becomes exacerbated under heat shock stress. Moreover, their response to heat shock is severely compromised, as evident from the insufficient upregulation of HSP83 and HSP70 mRNAs in *c14dm^−^ (*Figure 4A–B). Heat shock proteins are known to be induced in response to heat and other stress to increase cell survival under adverse conditions [48,49]. They act as molecular chaperones that facilitate the correct folding or degradation of misfolded proteins [50]. In addition, heat shock proteins in trypanosomatids are functionally adapted to a parasitic lifestyle, including resistance to the temperature changes and essential for the differentiation of parasites [51,52,53]. Our results suggest that the inability of *c14dm^−^* to cope with stress is, at least in part, due to dysregulation of gene expression.

It is known that a high level of oxidative stress can contribute to increased RNA degradation and reduced translation [54,55,56]. We have demonstrated earlier that *c14dm^−^* mutant displays mitochondrial dysfunction and elevated levels of oxidative stress [23]. However, the antioxidant treatment of the mutant with L-glutathione did not restore RNA levels (Figure 5). Moreover, the induction of oxidative stress in the mitochondrion of WT cells did not lead to RNA reduction. Thus, our data indicate that oxidative stress is not the main cause of RNA instability in *c14dm^−^*.

**Figure 10:**
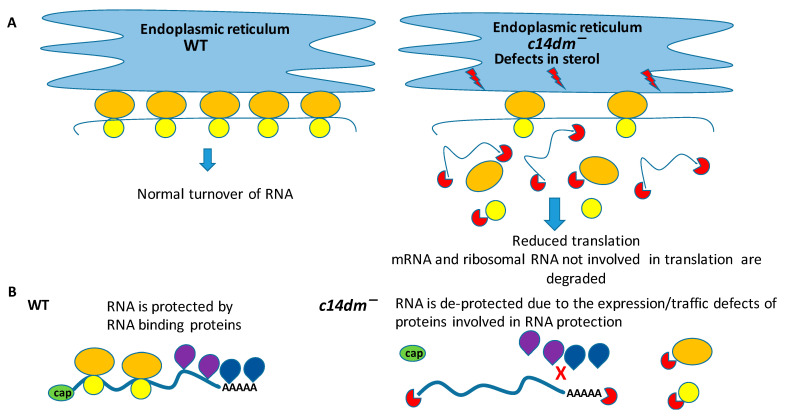
A model depicting the putative mechanism of RNA reduction in *c14dm^−^*. (**A**) Sterol synthesis defects may alter the lipid content of the ER and reduce ribosome recruitment, causing degradation of rRNA and mRNA not involved in translation. (**B**) Alternatively, defects in sterol synthesis may cause traffic defects preventing proteins involved in RNA protection from reaching and protecting RNA. Lightning bolts in red depict defects in sterol. Small (yellow) and large (orange) ovals represent small and large subunits of ribosomes, respectively. mRNA is shown as a blue line. Enzymes involved in RNA degradation are shown as partial red circles. RNA-binding proteins are shown in blue and purple.

C14DM is primarily localized in the ER [22]. The lack of endogenous sterols and accumulation of abnormal, C-14-methylated intermediates may lead to defects not only in the PM but also in the ER. Rough ER contains ribosomes actively involved in translation [57,58]. We hypothesize that impairment in sterol production leads to significant defects in the ER (Figure 10) and reduced recruitment of ribosomes to ER where the majority of translation occurs [37]. As a result, rRNA and mRNA not involved in translation are subjected to degradation. Our polysome profiling data demonstrate that monosome and light and heavy polysome peaks are lower in *c14dm^−^* mutant, supporting an overall reduced engagement in translation (Figure 7). Moreover, ER staining with BiP, an ER marker, is substantially reduced (by ~30%) during heat shock, suggesting defective ER in the mutant (Figure 9). It is possible that ribosomes cannot attach sufficiently due to the alteration of sterol content in the ER. It leads to reduced translation and RNA degradation, as shown in our proposed model (Figure 10A).

However, we cannot rule out other possibilities. Sterol defects in other organisms are known to affect not only membrane permeability and fluidity but also the localization of membrane-bound proteins and protein transport [59]. Therefore, it is possible that defects in sterol synthesis may compromise the expression/trafficking of RNA-stabilizing proteins, thus reducing their ability to reach and protect RNA (Figure 10B).

## 5. Conclusions

The role of lipids in controlling gene expression is not sufficiently understood not only in trypanosomatids but also in other eukaryotic organisms, including mammals. In the past, lipids have been mostly viewed as components of membranes and energy storage molecules. However, during the last few decades, a substantial amount of research revealed the importance of lipids as signaling molecules regulating many processes and gene expression pathways in the cell. Sphingolipids and their metabolites, such as ceramides, sphingosine and sphingosine-1-phosphate (S1P), regulate cellular processes, including proliferation, differentiation and cell death [60,61,62]. The S1P acts extracellularly through cell surface receptors. It also has important intracellular targets involved in inflammation, cancer and neurodegenerative diseases [63,64]. Phospholipids are exceptionally important for cellular signaling, and they can modulate the activity of nuclear hormone transcription factors responsible for the tuning of genes involved in the metabolism [65]. Sterols are known to be involved in signaling as well. For example, cholesterol activates cytokine production and regulates inflammation in mammalian cells [66]. While sterols are known to serve as structural components of eukaryotic membranes and signaling molecules regulating certain pathways in the cell, they appear to play an additional unexpected role. Our study is the first of its kind and supports a novel role of sterol in RNA stability, mRNA translation and global regulation of gene expression. As a result, it shifts our traditional understanding of the role of lipids. For future studies, it will be very important to determine what mechanism is responsible for RNA instability caused by sterol defects. Do defects in ER and PM structures contribute to reduced RNA stability? Is the recruitment of RNA-binding proteins affected? Is ribosome recruitment to ER affected? More studies are needed to determine the role of sterols in the regulation of RNA levels and reveal the mechanism of global RNA destabilization caused by lipid imbalance. Finally, the identification of components of RNA degradation and protection machinery may provide us new drug targets, and their inhibition combined with sterol inhibition may provide a better strategy to treat leishmaniasis.

## Data Availability

The data presented in this study are available on request from the corresponding authors.

## Supplementary Materials

The following are available online at https://www.mdpi.com/article/10.3390/biomedicines9060696/s1, Figure S1: Ribosomal RNA in *c14dm^−^* degrades faster than in WT and add-back parasites, File S1: Primers used in RT-qPCR.
