# Supplementary material for: Unexpected Role of Sterol Synthesis in RNA Stability and Translation in Leishmania
Source: Biomedicines. 2021 Jun 19;9(6):696. doi: 10.3390/biomedicines9060696 (PMC8235615; doi:10.3390/biomedicines9060696)
Supplement: Supplementary file 1 [file biomedicines-09-00696-s001.zip › Supplementary Figure S1.pptx]

## Slide 1
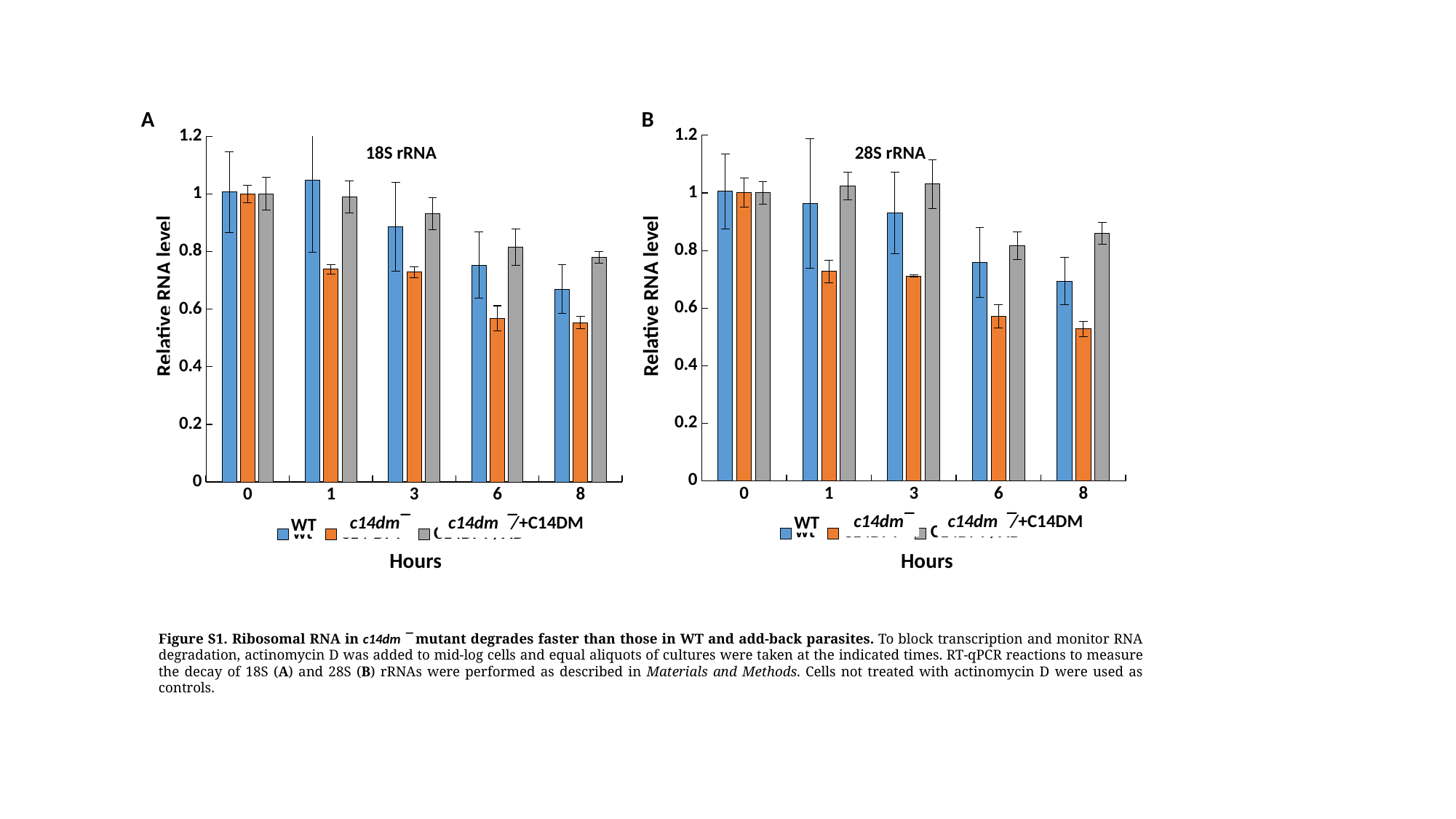

A
B
### Chart
| Category | wt | C14DM- | C14DM-/AB |
|---|---|---|---|
| 0 | 1.005896732670228 | 1.000865476235102 | 1.00051377635667 |
| 1 | 0.963154210007645 | 0.727865178535348 | 1.024333250228121 |
| 3 | 0.930736848125972 | 0.711540400513616 | 1.03067012602546 |
| 6 | 0.758217842381851 | 0.570718187754468 | 0.816048390437582 |
| 8 | 0.693380217149812 | 0.527851219929366 | 0.859511608426503 |
### Chart
| Category | wt | C14-DM- | C14DM-/AB |
|---|---|---|---|
| 0 | 1.006825318584539 | 1.000326041056651 | 1.001103645542651 |
| 1 | 1.047453850285828 | 0.738635181638071 | 0.990002628068891 |
| 3 | 0.887001655788604 | 0.729009147340452 | 0.931725576594667 |
| 6 | 0.753631439651073 | 0.568250211581502 | 0.816351889969324 |
| 8 | 0.66926917840059 | 0.553760254283141 | 0.780604401716434 |18S rRNA
28S rRNA
Relative RNA level
Relative RNA level
c14dm ̅
c14dm ̅/+C14DM
c14dm ̅
c14dm ̅/+C14DM
WT
WT
Hours
Hours
Figure S1. Ribosomal RNA in c14dm ̅ mutant degrades faster than those in WT and add-back parasites. To block transcription and monitor RNA degradation, actinomycin D was added to mid-log cells and equal aliquots of cultures were taken at the indicated times. RT-qPCR reactions to measure the decay of 18S (A) and 28S (B) rRNAs were performed as described in Materials and Methods. Cells not treated with actinomycin D were used as controls.
